# Supplementary material for: Genetic Diversity Analysis Reveals Potential of the Green Peach Aphid (Myzus persicae) Resistance in Ethiopian Mustard
Source: Int J Mol Sci. 2022 Nov 8;23(22):13736. doi: 10.3390/ijms232213736 (PMC9699141; doi:10.3390/ijms232213736)
Supplement: Supplementary file 1 [file ijms-23-13736-s001.zip › Table S1.pdf]

Table S1 75 Ethiopian Mustard accessions and their geographical origin used in this study

Acc. Code	Country of origin	Area of collection site	Acc. Code	Country of origin	Area of collection site
BC01	Ethiopia	Holetta	BC39	Ethiopia	—
BC02	Ethiopia	Holetta	BC40	Sweden	—
BC03	Ethiopia	Holetta	BC41	Sweden	—
BC04	Ethiopia	Holetta	BC42	Sweden	—
BC05	Ethiopia	Holetta	BC43	Sweden	—
BC06	Ethiopia	Holetta	BC44	Sweden	—
BC07	UK	—	BC45	Pakistan	—
BC08	Ethiopia	Shewa	BC46	Pakistan	—
BC09	Ethiopia	Hararghe	BC47	Spain	Córdoba
BC10	Ethiopia	Shewa	BC48	Spain	Córdoba
BC11	Ethiopia	Shewa	BC49	Sweden	—
BC12	Ethiopia	Kefa	BC50	Spain	Cordoba
BC13	Ethiopia	Kefa	BC51	—	—
BC14	Ethiopia	Kefa	BC52	—	—
BC15	Ethiopia	Kefa	BC53	—	—
BC16	Ethiopia	Kefa	BC54	—	—
BC17	Ethiopia	Gonder	BC55	—	—
BC18	Ethiopia	Gonder	BC56	Ethiopia	Hararghe
BC19	Ethiopia	Gonder	BC57	Ethiopia	Hararghe
BC20	Ethiopia	Gonder	BC58	Ethiopia	Hararghe
BC21	Ethiopia	Wello	BC59	Sao Tome and Principe	—
BC22	Ethiopia	Wello	BC60	—	—
BC23	Ethiopia	Wello	BC61	Ethiopia	Illubabor
BC24	Ethiopia	Welega	BC62	Ethiopia	—
BC25	Ethiopia	—	BC63	Ethiopia	Hararghe
BC26	Ethiopia	—	BC64	Tanzania	—
BC27	Ethiopia	—	BC65	Kenya	—
BC28	Ethiopia	Gonder	BC66	Tanzania	—
BC29	Ethiopia	Kefa	BC67	Zambia	—
BC30	Puerto Rico	—	BC68	Russian	—
BC31	Ethiopia	Shewa	BC69	Ethiopia	—
BC32	Ethiopia	Shewa	BC70	Ethiopia	—
BC33	Ethiopia	Shewa	BC71	Ethiopia	—
BC34	Ethiopia	Harer	BC72	Ethiopia	—
BC35	Ethiopia	Harer	BC73	Ethiopia	—
BC36	Ethiopia	Shewa	BC74	Ethiopia	—
BC37	Ethiopia	—	BC75	Pakistan	—
BC38	Ethiopia	—			

Note: BC01-BC06 was collected from Ethiopia Holetta Agricultural research Center. BC51-BC55 was collected from Oil Crops Research Institute (Chinese Academy of Agricultural Sciences). BC56-BC63 was collected from Professor Jun Zou's Laboratory (Huazhong Agricultural University). Others were collected from U.S. National

Plant Germplasm System.
