# Supplementary material for: Genetic Diversity Analysis Reveals Potential of the Green Peach Aphid (Myzus persicae) Resistance in Ethiopian Mustard
Source: Int J Mol Sci. 2022 Nov 8;23(22):13736. doi: 10.3390/ijms232213736 (PMC9699141; doi:10.3390/ijms232213736)
Supplement: Supplementary file 1 [file ijms-23-13736-s001.zip › Table S2.pdf]

Table S2 Genetic diversity analysis of qualitative traits for 75 Ethiopian Mustard accessions

| Morphological traits | Minimum value | Maximum value | Mean value | Standard deviation | Coefficient of variation | Genetic diversity index H’ |
|---|---|---|---|---|---|---|
| Plant height (cm) | 10.30 | 23.73 | 17.13 | 2.93 | 17.12% | 2.02 |
| Plant width (cm) | 20.50 | 39.70 | 30.05 | 3.96 | 13.19% | 2.04 |
| Plant length (cm) | 17.40 | 35.23 | 26.99 | 4.10 | 15.19% | 2.06 |
| Number of leaves | 7.00 | 16.33 | 10.96 | 1.63 | 14.83% | 2.00 |
| Petiole length (cm) | 4.60 | 13.00 | 8.37 | 1.61 | 19.18% | 1.96 |
| Petiole width (cm) | 0.32 | 1.02 | 0.69 | 0.13 | 18.12% | 2.00 |
| Petiole thick (cm) | 0.28 | 0.69 | 0.46 | 0.07 | 15.42% | 2.01 |
| Lamina length (cm) | 11.37 | 24.97 | 18.15 | 2.65 | 14.61% | 1.95 |
| Lamina width (cm) | 4.80 | 11.03 | 8.28 | 1.36 | 16.44% | 2.01 |
| Seed diameter (mm) | 1.38 | 2.16 | 1.76 | 0.16 | 8.85% | 2.06 |
