# Supplementary material for: Genetic Diversity Analysis Reveals Potential of the Green Peach Aphid (Myzus persicae) Resistance in Ethiopian Mustard
Source: Int J Mol Sci. 2022 Nov 8;23(22):13736. doi: 10.3390/ijms232213736 (PMC9699141; doi:10.3390/ijms232213736)
Supplement: Supplementary file 1 [file ijms-23-13736-s001.zip › Table S3.pdf]

Table S3 Genetic diversity analysis of quality traits for 75 Ethiopian Mustard accessions

Morphological traits	Frequency distribution (%)										Coefficient of variation	Genetic diversity index H’
	1	2	3	4	5	6	7	8	9	10		
Plant type	69.33	25.33	2.67	2.67	0	0	0	0	0	0	48.73%	0.80
Leaf type	100.00	0	0	0	0	0	0	0	0	0	0.00%	0.00
Leaf shape	16.00	37.33	29.33	9.33	2.67	1.33	4.00	0	0	0	51.65%	1.53
Leaf blade blistering	8.00	60.00	26.67	5.33	0	0	0	0	0	0	30.22%	1.02
Pinnately compound leaf	2.67	26.67	6.67	64.00	0	0	0	0	0	0	28.94%	0.92
Leaf hairiness	100.00	0	0	0	0	0	0	0	0	0	0.00%	0.00
Leaf apex shape	61.33	37.33	1.33	0	0	0	0	0	0	0	37.13%	0.73
Leaf margin wavy	9.33	37.33	53.33	0	0	0	0	0	0	0	27.15%	0.92
Leaf crack	100.00	0	0	0	0	0	0	0	0	0	0.00%	0.00
Leaf wax powder	10.67	50.67	24.00	14.67	0	0	0	0	0	0	35.96%	1.21
Leaf color	32.00	54.67	13.33	0	0	0	0	0	0	0	35.92%	0.96
Vein clarity	1.33	98.67	0	0	0	0	0	0	0	0	5.81%	0.07
Petiole color	30.67	40.00	29.33	0	0	0	0	0	0	0	39.25%	1.09
