# Supplementary material for: Genetic Diversity Analysis Reveals Potential of the Green Peach Aphid (Myzus persicae) Resistance in Ethiopian Mustard
Source: Int J Mol Sci. 2022 Nov 8;23(22):13736. doi: 10.3390/ijms232213736 (PMC9699141; doi:10.3390/ijms232213736)
Supplement: Supplementary file 1 [file ijms-23-13736-s001.zip › Table S5.pdf]

Table S6 Identification of seed quality characters for 75 Ethiopian Mustard accessions

Seed character	Minimum value	Maximum value	Mean value	Standard deviation	Coefficient of variation
Oil (%)[1]	24.30	41.70	29.22	2.85	10%
Glucosinolates (μmol/g)[1]	98.10	154.30	122.27	10.57	9.0%
Protein (%)[1]	23.20	33.55	29.69	1.97	7.0%
Moisture (%)[1]	2.10	5.45	3.63	0.72	20%
Erucic acid (%)[2]	0.00	25.45	7.49	4.98	66%
Oleic acid (%)[2]	55.40	70.30	63.70	2.98	5.0%
Linolenic acid (%)[2]	4.65	11.45	7.34	0.94	13%
Saturated FA (%)[2]	5.40	7.35	6.51	0.32	5.0%

[1] Content in seeds. [2] Content in oil.
