# Supplementary material for: Genetic Diversity Analysis Reveals Potential of the Green Peach Aphid (Myzus persicae) Resistance in Ethiopian Mustard
Source: Int J Mol Sci. 2022 Nov 8;23(22):13736. doi: 10.3390/ijms232213736 (PMC9699141; doi:10.3390/ijms232213736)
Supplement: Supplementary file 1 [file ijms-23-13736-s001.zip › Table S7.pdf]

Table S7 Identification and statistics of aphid resistance screening of 75 Ethiopian mustard

Accession code	7DAI	14DAI	21DAI	Aphid number ratio	Evalution
BC01	67.00±27.78	310.43±145.05	733.87±23.68	1.51	HS
BC02	53.13±49.72	169.87±91.92	480.73±99.64	0.98	LS
BC05	55.60±23.12	165.60±34.37	409.53±86.25	0.84	LR
BC06	29.13±12.35	232.13±74.99	517.53±58.24	1.06	LS
BC07	40.40±20.99	193.47±114.93	458.73±67.32	0.94	LS
BC08	21.23±1.48	84.80±25.12	405.27±129.60	0.83	LR
BC09	44.87±16.98	123.68±39.55	364.80±134.42	0.75	LR
BC10	60.95±38.20	249.37±188.44	458.25±158.50	0.94	LS
BC11	64.40±33.18	311.80±144.01	530.07±129.28	1.09	LS
BC12	36.83±15.01	177.70±90.01	349.23±33.89	0.71	LR
BC13	17.93±7.60	96.46±61.18	216.15±32.14	0.44	MR
BC14	59.13±10.82	243.13±62.64	447.00±113.79	0.92	LS
BC15	36.98±16.54	142.69±76.82	349.40±52.89	0.72	LR
BC16	36.69±26.48	115.31±50.27	327.13±53.07	0.67	LR
BC17	64.07±32.62	264.75±130.43	418.73±57.88	0.86	LR
BC18	38.07±9.55	128.40±15.07	287.93±54.33	0.59	MR
BC19	30.98±19.64	142.44±86.34	604.53±297.47	1.24	MS
BC20	87.69±2.75	394.27±55.14	605.13±125.09	1.24	MS
BC21	19.80±4.54	101.07±51.50	325.80±21.53	0.67	LR
BC22	21.33±5.14	132.73±60.23	602.40±76.83	1.23	MS
BC23	28.56±10.50	110.80±33.85	364.96±126.08	0.75	LR
BC24	53.13±17.33	261.90±23.32	595.67±179.25	1.22	MS
BC25	36.07±16.87	214.93±77.04	747.00±74.94	1.53	HS
BC26	27.13±2.84	126.33±50.76	390.32±98.60	0.80	LR
BC27	35.42±4.14	62.50±47.38	316.40±69.94	0.65	LR
BC28	35.50±17.76	166.47±73.60	611.60±69.81	1.25	MS
BC29	23.53±11.81	124.80±62.45	474.80±161.24	0.97	LS
BC30	35.42±4.14	101.13±55.87	310.00±36.00	0.63	LR
BC31	35.50±17.76	83.40±43.96	484.73±12.20	0.99	LS
BC32	23.53±11.81	142.36±5.80	593.97±107.12	1.22	MS
BC33	29.00±3.61	265.00±40.11	676.00±106.66	1.38	MS
B34	24.75±9.43	111.87±58.59	562.45±157.55	1.15	LS
BC35	16.69±5.88	74.78±47.10	308.09±75.15	0.63	LR
BC36	13.20±2.31	126.95±61.48	451.40±246.23	0.92	LS
BC37	34.27±27.12	86.52±72.06	291.44±58.47	0.60	MR
BC39	11.73±3.42	73.37±45.58	297.32±103.48	0.61	LR
BC40	18.33±11.42	138.73±90.86	366.40±111.31	0.75	LR
BC41	12.98±4.81	63.29±32.80	435.90±52.86	0.89	LR
BC42	35.70±12.56	135.93±104.91	558.90±280.37	1.14	LS
BC43	24.61±8.60	108.53±58.22	493.92±243.35	1.01	LS
BC44	30.80±5.96	68.38±51.46	406.27±23.98	0.83	LR
BC45	25.07±11.57	94.92±29.60	407.75±30.36	0.83	LR

BC47	20.73±11.89	95.69±47.73	260.50±127.65	0.53	MR
BC48	31.04±15.59	134.69±77.59	606.80±196.77	1.24	MS
BC49	23.55±3.04	119.65±34.54	385.65±182.43	0.79	LR
BC51	9.87±3.44	59.07±17.17	257.67±30.13	0.53	MR
BC52	35.32±13.29	254.39±32.48	605.36±124.34	1.24	MS
BC53	39.13±5.61	306.13±83.82	575.52±106.94	1.18	LS
BC54	67.07±16.39	385.85±110.83	544.93±88.22	1.12	LS
BC55	38.00±15.52	301.73±97.54	530.93±21.04	1.09	LS
BC56	27.73±19.30	173.40±75.28	606.47±89.77	1.24	MS
BC57	45.27±10.03	267.07±58.61	519.37±59.30	1.06	LS
BC58	9.07±2.25	179.20±46.22	441.87±61.88	0.90	LR
BC59	37.20±25.49	224.60±90.35	502.70±51.23	1.03	LS
BC60	57.80±17.51	474.33±29.74	766.35±56.42	1.57	HS
BC61	58.67±17.98	330.20±74.16	567.27±20.28	1.16	LS
BC62	47.74±1.72	247.28±58.05	517.11±52.04	1.06	LS
BC63	29.13±6.47	293.67±42.47	583.20±80.13	1.19	LS
BC65	70.33±24.54	336.02±66.84	559.00±31.20	1.14	LS
BC66	57.73±5.54	295.80±118.87	612.33±98.11	1.25	MS
BC67	31.00±12.00	320.00±138.73	559.67±84.21	1.15	LS
BC68	22.87±6.80	259.00±66.06	693.8±162.10	1.42	MS
BC70	43.60±25.70	270.20±34.30	569.43±194.40	1.17	LS
BC71	45.68±18.08	277.00±28.81	630.06±30.49	1.29	MS
BC72	43.87±6.33	344.33±91.08	559.40±83.22	1.15	LS
BC73	29.40±6.92	224.87±75.09	464.27±57.57	0.95	LS
BC74	32.58±16.09	203.13±8.43	645.38±45.39	1.32	MS
BC75	40.53±13.08	268.00±76.09	614.45±71.96	1.26	MS

Note: HR, Highly resistant; MR, Moderately resistant; LR, Low resistant; LS，Low Susceptible; MS, Moderately Susceptible; HS，Highly susceptible. 7/14/21D means on day 7/14/21 after inoculation. Data are means ± SE.
