# Supplementary figures and images for: Genetic Diversity Analysis Reveals Potential of the Green Peach Aphid (Myzus persicae) Resistance in Ethiopian Mustard
Source: Int J Mol Sci. 2022 Nov 8;23(22):13736. doi: 10.3390/ijms232213736 (PMC9699141; doi:10.3390/ijms232213736)

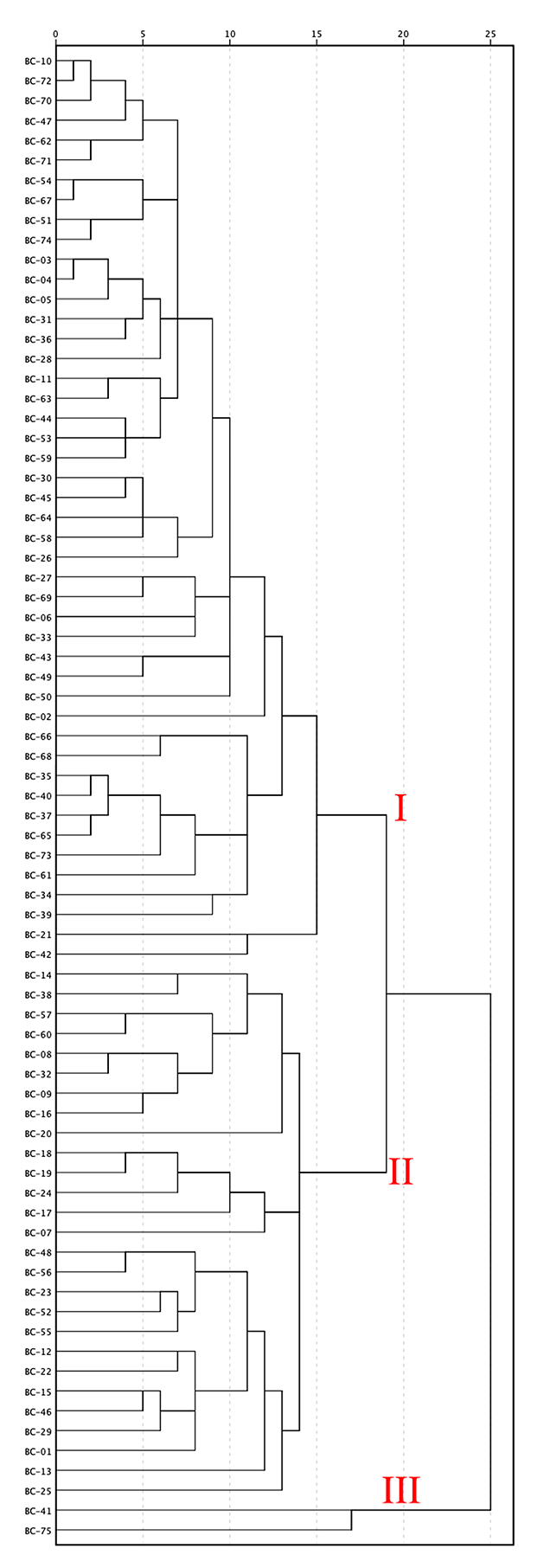

Supplement: Supplementary file 1 [file ijms-23-13736-s001.zip › Figure S1.tif]

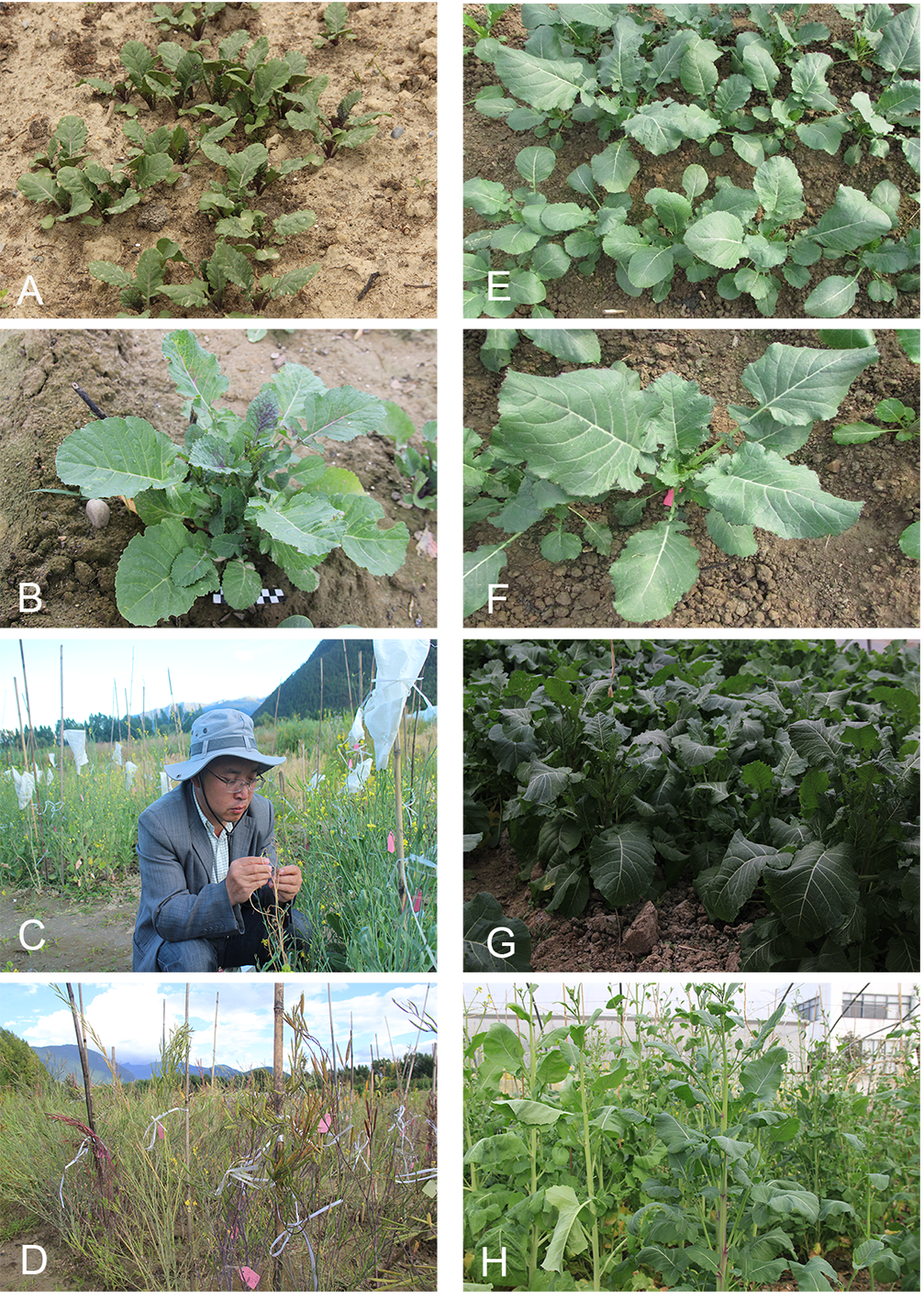

Supplement: Supplementary file 1 [file ijms-23-13736-s001.zip › Figure S2.tif]

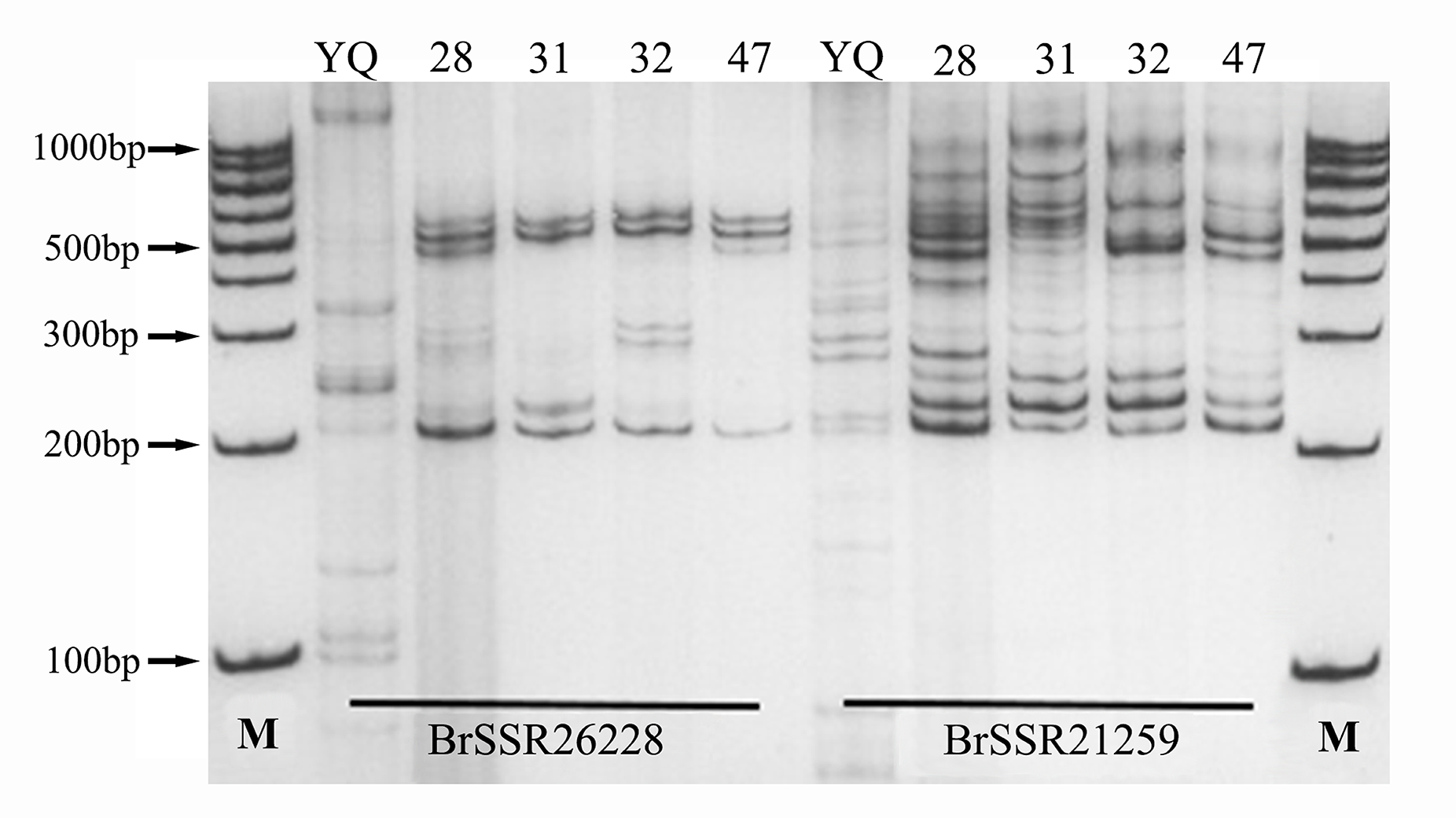

Supplement: Supplementary file 1 [file ijms-23-13736-s001.zip › Figure S3.tif]

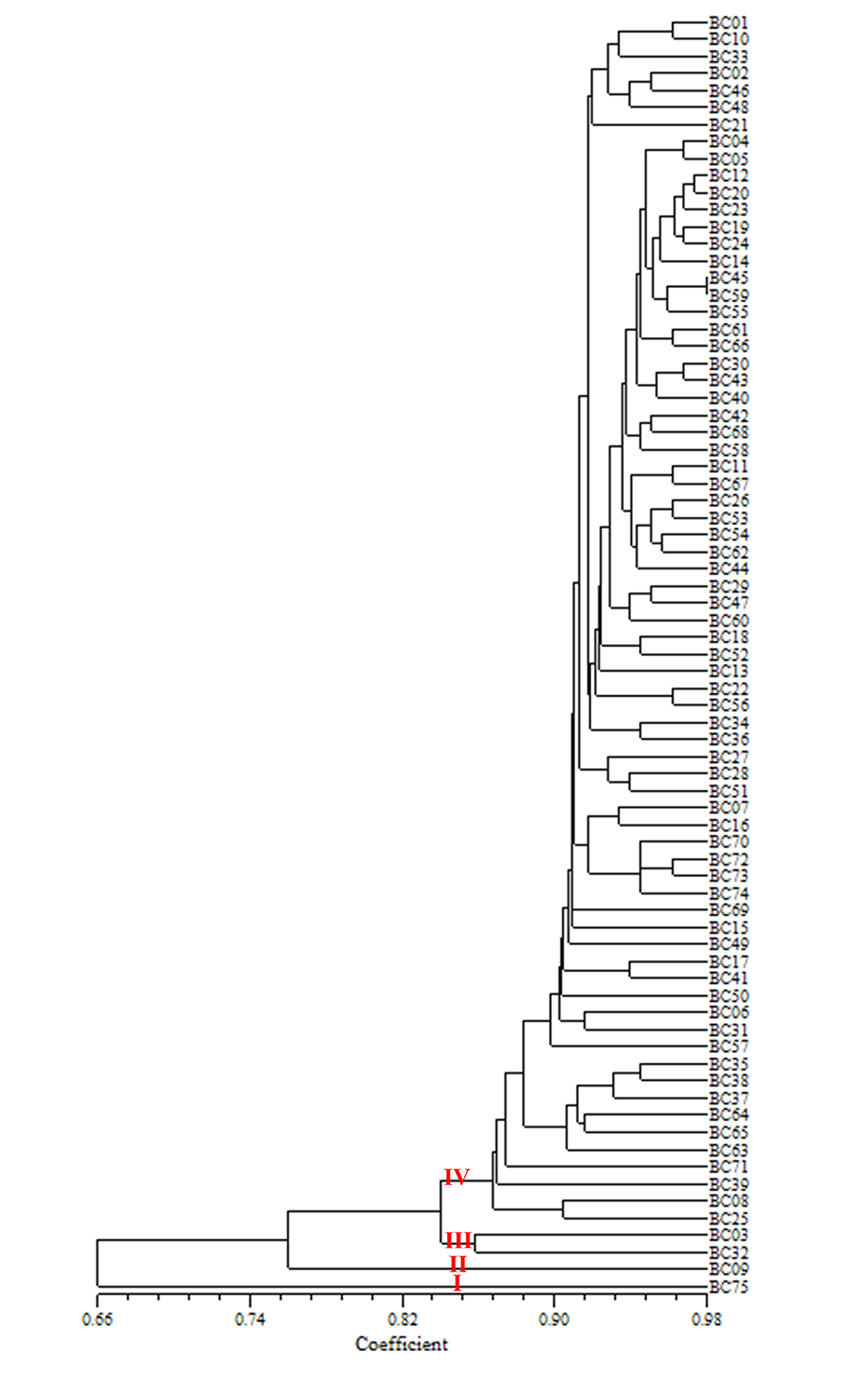

Supplement: Supplementary file 1 [file ijms-23-13736-s001.zip › Figure S4.tif]

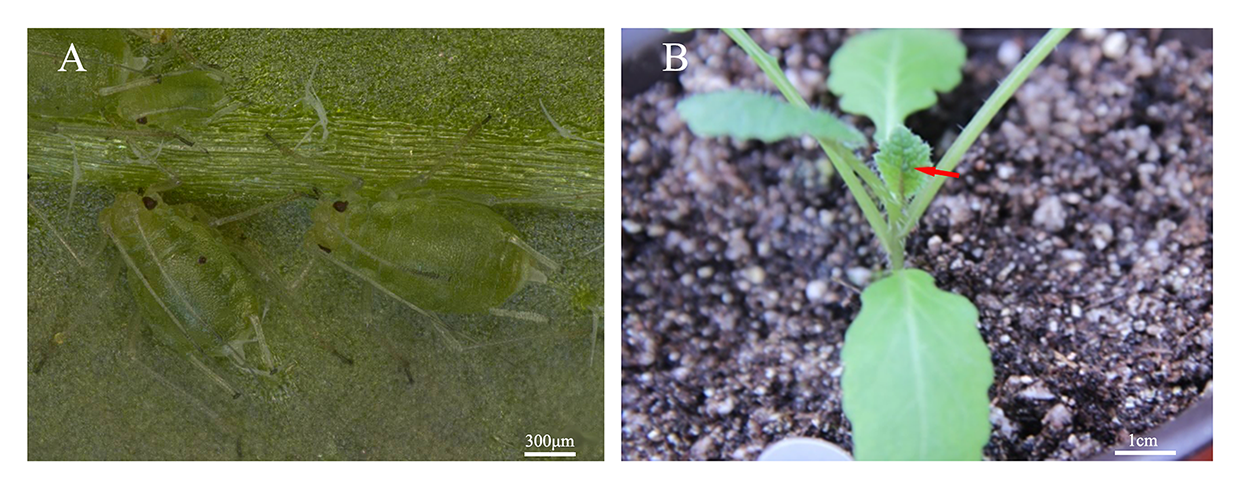

Supplement: Supplementary file 1 [file ijms-23-13736-s001.zip › Figure S5.tif]

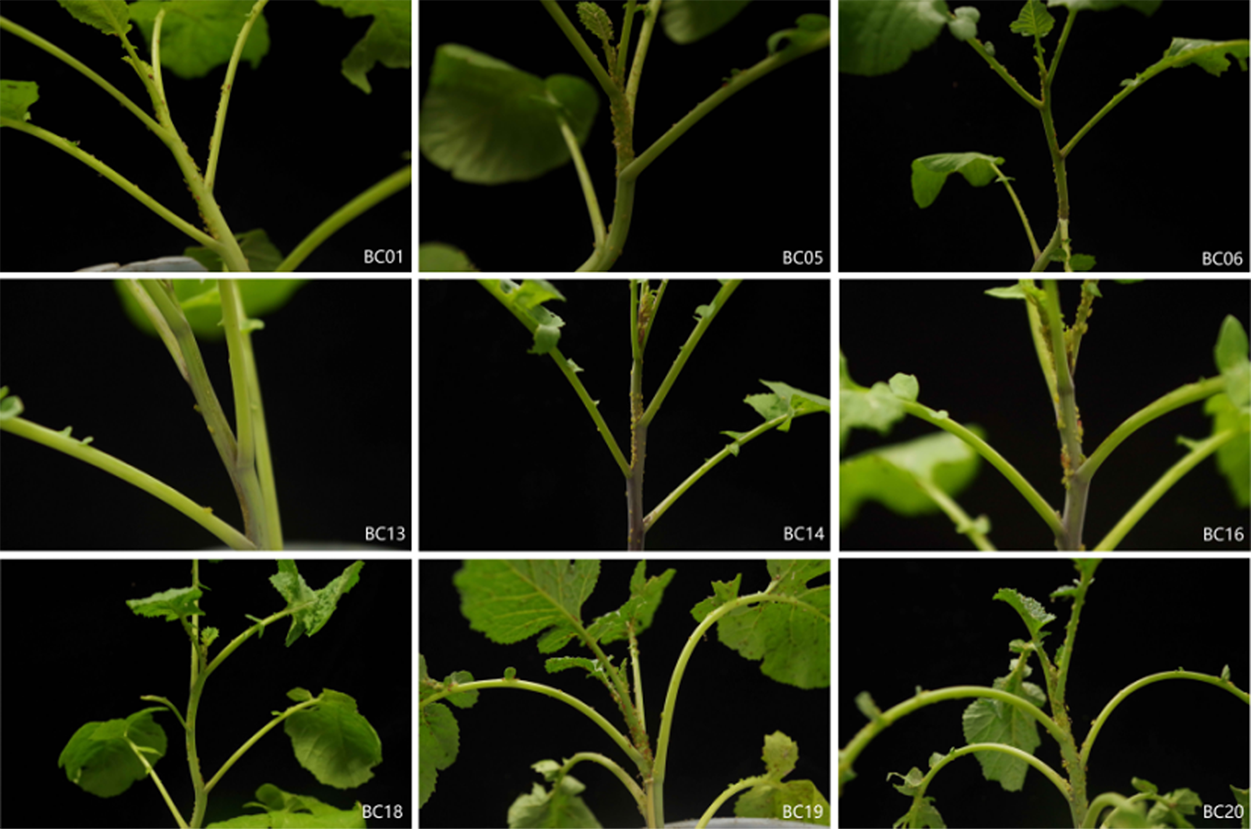

Supplement: Supplementary file 1 [file ijms-23-13736-s001.zip › Figure S6.tif]

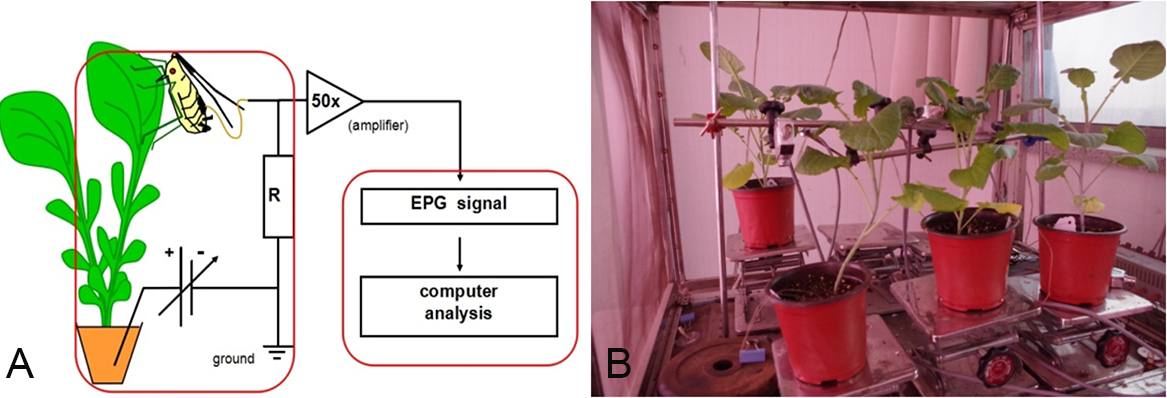

Supplement: Supplementary file 1 [file ijms-23-13736-s001.zip › Figure S7.tif]

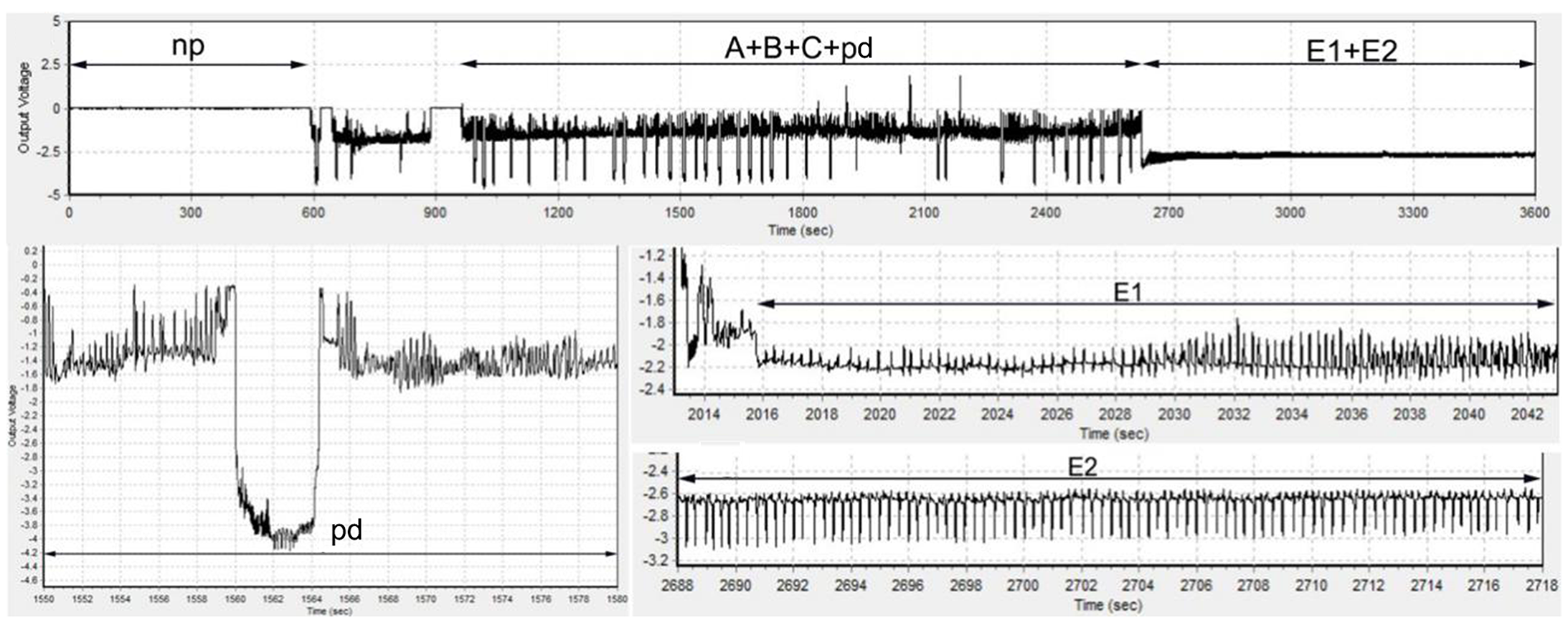

Supplement: Supplementary file 1 [file ijms-23-13736-s001.zip › Figure S8.tif]

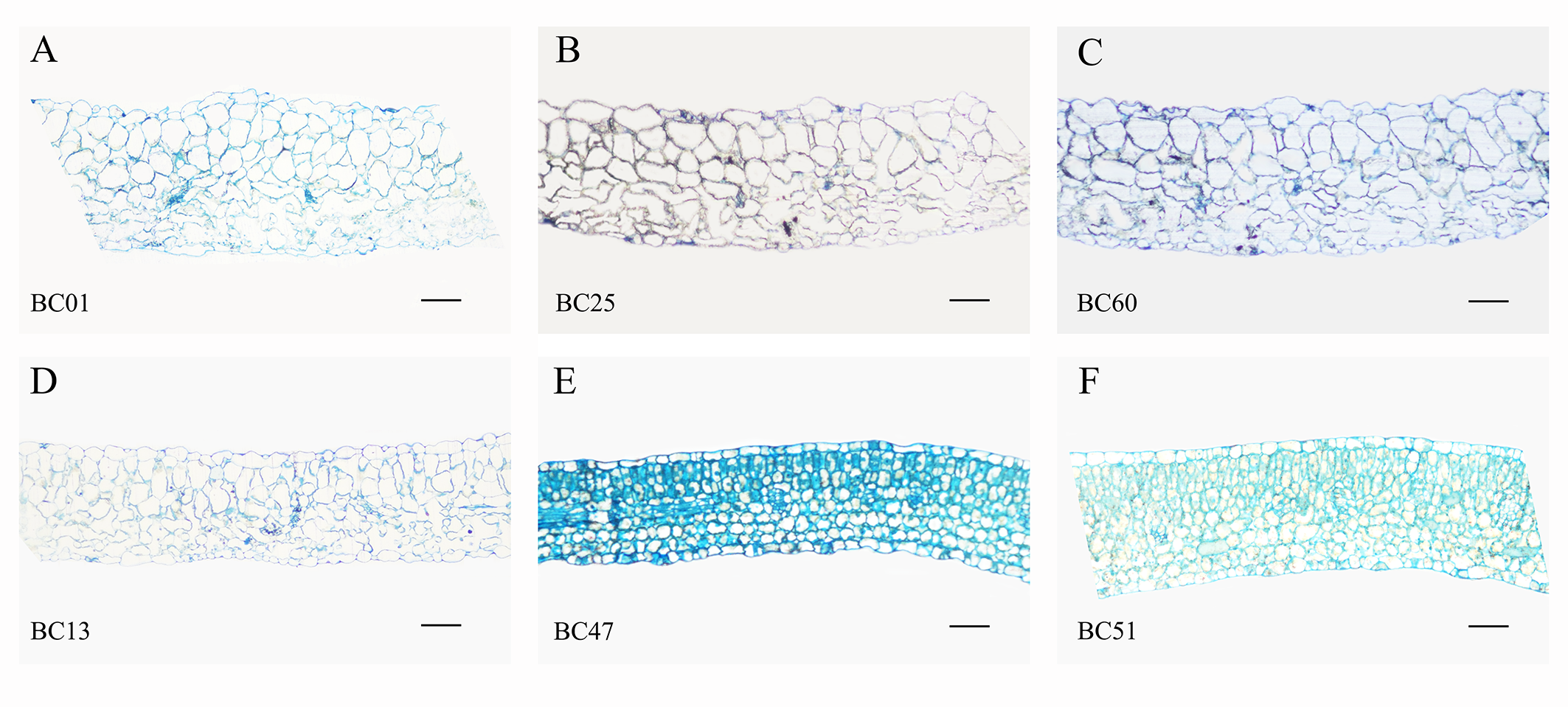

Supplement: Supplementary file 1 [file ijms-23-13736-s001.zip › Figure S9.tif]
